# 

**Published:** 1919-05

**Authors:** 


### Old Jokes That We Have Met.

FIGURATIVELY SPEAKING:—Dr. Wiley tells the following story: Sleepily, after a night off, a certain interne hastened to his hospital ward. The first patient was a stout old Irishman.

"How goes it?" he inquired.

"Faith, it'sh me breathin', doctor, I can't get me breath at all, at all."

"Why, your pulse is normal. Let me examine the lungaction,'' replied the doctor, kneeling beside the cot and laying his head on the ample chest.

''Now, let's hear you talk,'' he continued, closing his eyes and listening.

''What'll Oi be sayin', doctor?''

''Oh, say anything. Count one, two, three and up,'' murmured the interne, drowsily.

''Wan, two, three, four, five, six,'' began the patient. When the young doctor, with a start, opened his eyes, he was counting huskily. ''Tin hundred an' sixty-nine, tin hundred an' sivinty, tin hundred an' sivinty-wan.''—Christian Register.

---

INSANITARY.

Jack and Jill went up the hill,
Like dutiful son and daughter.
Now Jack has typhoid, Jill is ill—
They didn't boil the water.

J. W. Foley.

---

BUT HE GETS IT:—''Dr. Blank frequently accepted no fees from his patients.''

''You don't say so?''

''He settles with the heirs.''—Boston Transcript.

---

MEDICAL IGNORANCE—Dr. Simon Flexner of the Rockefeller Institute, who has just received one of the French Government's highest decorations, told a story at a New York dinner.

''The medical ignorance of some people is staggering,'' he said. ''I know an X-ray specialist who got a letter from a Middle Western farmer the other day. The farmer wrote:

'' 'Dear Sir: I have had a nail in my thorax for seventeen years. I am too busy to come to New York, but want you to come down here to Paris Corners with your rays, as my case will be worth your while. If you do not find time to come, send a dozen rays boxed, by express, with instruction card, and I will try to work same myself.'

''The X-ray specialist wrote back to the farmer of Paris Corners:

'' 'Dear Friend: I regret to say that business engagements prevent my trip to Paris Corners, and I am unfortunately out of rays just now. If you cannot come to New York, send me your thorax by parcel post, and we will see what can be done.' ''
—Washington Star.

